# Systematic Pharmacology and GEO Database Mining Revealed the Therapeutic Mechanism of Xuefu Zhuyu Decoration for Atherosclerosis Cardiovascular Disease

**DOI:** 10.3389/fcvm.2020.592201

**Published:** 2020-12-03

**Authors:** Bin Liang, Yang Xiang, Xiaokang Zhang, Chen Wang, Bingyu Jin, Yue Zhao, Fang Zheng

**Affiliations:** Center for Gene Diagnosis, Zhongnan Hospital of Wuhan University, Wuhan, China

**Keywords:** systematic pharmacology, Xuefu Zhuyu decoration, atherosclerosis cardiovascular disease, therapeutic mechanism, GEO database

## Abstract

**Background:** Xuefu Zhuyu decoration (XFZYD), as a traditional Chinese compound recipe, has been used to treat atherosclerosis cardiovascular disease (ASCVD) for thousands of years in China, but its effective compounds and underlying treatment molecular mechanism remains promiscuous, which severely limits its clinical application.

**Methods:** The effective components and their targets of XFZYD were predicted and screened based on the Traditional Chinese Medicine System Pharmacology (TCMSP) database. The candidate therapeutic targets of ASCVD were screened by Pharmacogenomics Knowledgebase (PharmGKB) and Comparative Toxicogenomics Database (CTD). Kyoto Encyclopedia of Genes and Genomes (KEGG) pathway analyses for target proteins were performed using the Database for Annotation, Visualization and Integrated Discovery (DAVID) database. Differentially expressed genes were identified using the GEO2R online tool. Molecular docking was performed by Schrodinger software. To assess the efficacy of the prediction, human umbilical vein endothelial cells (HUVECs) treated with the effective compound of XFZYD were used as the *in vitro* model.

**Results:** A total of 108 effective compounds (including quercetin) and 137 candidate therapeutic targets were identified. Analyzing the relationships among effective compounds, candidate therapeutic targets, and signaling pathways, the therapy mechanisms of XFZYD were mainly reflected in the protection of vascular endothelium, anti-inflammatory, antioxidant stress, etc. Accordingly, we found the effective compound of XFZYD (quercetin) decreased intracellular adhesion molecule 1 (ICAM-1) and vascular cell adhesion molecule 1 (VCAM-1) expressions and pro-inflammatory cytokines in HUVECs treated with lipopolysaccharide (LPS), and reduced the adhesion function of HUVECs with monocytes. The inhibitor of the predicted target protein (PTGS2) could further reduce the expressions of VCAM-1, ICAM-1, and TNF-α induced by LPS, and inhibit the adhesion function of HUVECs with monocytes, while PTGS2 agonists partially counteracted the protective effect of quercetin.

**Conclusions:** In this study, the effective components and potential therapeutic targets of XFZYD for ASCVD treatment were explored from the perspective of systemic pharmacology. The effective component quercetin was verified to protect endothelial cells by reducing endothelial inflammatory response and impeding the attachment of monocytes against the predicted therapeutic target PTGS2.

## Introduction

Atherosclerosis cardiovascular disease (ASCVD) was a systemic disease based on atherosclerosis, becoming a leading killer worldwide due to the high morbidity and mortality (1). Atherosclerosis is a pathological status characterized by fibrogenesis, chronic inflammation, lipid accumulation, and vascular wall immunity disorders (2, 3). As atherosclerotic plaques develop into advanced stages, brittle plaques tend to rupture (4), leading to acute cardiovascular events such as ischemic stroke and myocardial infarction.

At present, clinical medications ideas for ASCVD mainly focus on correcting of atherogenic dyslipidemia and platelet aggregation. The clinical efficacy of statins and aspirin has been well-established (5, 6). Although current atherosclerosis medications can partially relieve the symptoms of ASCVD patients, gastrointestinal reactions, liver and kidney damage, etc. occur constantly, due to individual differences, adverse effects of medicine, or usage without doctor's prescription, which greatly disturb the therapeutic efficacy.

Traditional Chinese Medicine (TCM) plays an important role in the adjuvant treatment of ASCVD (7). Compared with single drug prescriptions, TCM compound recipe has the characteristics of multicomponent, multitarget, and multipathway interaction. Xuefu zhuyu decoration (XFZYD), a famous herbal remedy, has been used to relieve symptoms in patients with ASCVD for thousand years in China with few adverse events (8). XFZYD is composed of 11 herbs: *Semen Persicae, Flos Carthami, Rhizoma Chuanxiong, Radix Angelicae Sinensis, Radix Paeoniae Rubra, Radix Rehmanniae, Fructus Aurantii, Radix Bupleuri, Radix Platycodonis, Radix Achyranthis Bidentatae*, and *Radix Glycyrrhizae*, and quercetin was shown to be one of the major components of XFZYD evaluated using ultrahigh-performance liquid chromatography with hybrid ion trap time-of-flight mass spectrometry (UHPLC-ESI-IT-TOF-MS) (9). These herbs have been shown to have antiatherosclerosis properties; for example, *Rhizoma Chuanxiong* was demonstrated to prevent atherosclerosis as well as ischemia–reperfusion injury in clinical trials (10). *Radix Glycyrrhizae* possessed important antioxidant activity and protective effect against the human lipoprotein oxidative system (11). Moreover, this formula has been proven reliable and effective for curing ASCVD (8, 12) and its risk factors such as hyperlipidemia (13) and hypertension (14). It has been documented to function as an anti-inflammatory agent by inhibiting the PI3K-AKT-mTOR pathway (15, 16) to increase coronary blood flow, to improve the cardiac microcirculation, to accommodate blood lipids (13), and to prevent platelet aggregation, maintaining vessel growth in physiological or repair range to avoid angiogenesis in the atheromatous plaque (17).

Despite increasing number of researches into the cardioprotective effects of XFZYD, the characterization of its effective compounds and the exact mechanisms underlying its therapeutic action are not fully understood. In the present study, the effective components, potential therapeutic targets, and therapeutic pathways of XFZYD for ASCVD were described, and the therapeutic mechanism of quercetin and therapeutic targets PTGS2 on human umbilical vein endothelial cells (HUVECs) were verified *in vitro*.

## Materials and Methods

### Identification of Effective Compounds of XFZYD

The effective components in XFZYD were identified from the Traditional Chinese Medicine System Pharmacology (TCMSP, http://lsp.nwu.edu.cn/browse.php) database. The database provides comprehensive information about ingredients in herbs including chemical structure, oral bioavailability (OB), half-life (HL), drug likeness (DL), drug targets, etc. The pharmacokinetic properties including absorption, distribution, metabolism, and excretion (ADME) are important contributors for bioactivities of drugs. In this study, three ADME-related parameters including OB ≥30%, HL ≥4, and DL ≥0.18 were employed to identify the potential effective compounds in XFZYD. As recommended by TCMSP, the compounds with OB ≥30% and HL ≥4 have good absorption and slow metabolism after oral administration, while the compounds with DL ≥0.18 were chemically suitable for drug development.

### Prediction of Compound-Related Targets

The compound-related targets were predicted depending on chemical similarities and pharmacophore models via the TCMSP databases. TCMSP compound data were obtained from databases such as DrugBank, HIT, TTD, Pharmacogenomics Knowledgebase (PharmGKB), etc. All the targets obtained above were standardized as gene names and UniProt IDs by searching from UniprotKB database with “*Homo sapiens*” species.

### Identification of ASCVD-Related Therapeutic Targets

These ASCVD-related therapeutic targets were mined from two databases including PharmGKB and Comparative Toxicogenomics Database (CTD). The key words were “atherosclerosis,” “coronary heart disease,” “angina,” “acute coronary syndrome,” “stroke,” “transient ischemic attacks,” and “peripheral arterial disease.” All the targets in PharmGKB and the top 200 targets in CTD based on inference score were selected, and the obtained targets were standardized as gene names and UniProt IDs by searching from UniprotKB database with “*Homo sapiens*” species.

### Network Construction and Topological Analysis

The compound–target network of XFZYD were constructed by Cytoscape v3.7.1 software, which is a tool for analysis and visualization of the biological network (18). The topological analysis was performed by the Network Analyzer module of Cytoscape software. According to the topology of network, degree centrality (DC), betweenness centrality (BC), and closeness centrality (CC) are the most important parameters for measuring the criticality of a node in the network, as well as the important index for new drug discovery and target prediction.

### KEGG Pathway Enrichment Analysis

The KEGG pathway enrichment analysis was carried out using the Database for Annotation, Visualization and Integrated Discovery (DAVID) database, which is an online biological knowledgebase and an analytic tool to extract biological information about gene functional classification, functional annotation, and enriched pathways (19). KEGG pathways with *P* < 0.05 were considered statistically significant.

### GEO Database Validation

Peripheral blood RNA expression profiles of three atherosclerosis patients and three controls were obtained from GSE71226, and the differentially expressed genes (DEGs) in GSE71226 were identified using GEO2R (https://www.ncbi.nlm.nih.gov/geo/geo2r/). *P* < 0.05 and |logFC| ≥ 1.5 were the screening criteria.

### Binding Capacity Between Effective Compounds and Key Targets by Molecular Docking

The X-ray crystal structures of the candidate therapeutic targets were taken from the RCSB PDB database, and all 3D structures of these components were obtained from the PubChem database. Molecular docking was performed by Schrodinger software. The compounds and target proteins were input and pretreated to perform the molecular docking command, and finally, the docking score was obtained. The magnitude of the absolute value of docking score is proportional to the strength of bonding.

### Cell Culture and Treatment

HUVECs were supplemented with Dulbecco's modified Eagle medium (DMEM, Gibco) containing 10% fetal bovine serum (Gibco) and 1% penicillin/streptomycin (Gibco) in a CO_2_ incubator (5% CO_2_ at 37°C). HUVECs were stimulated with lipopolysaccharide (LPS) (10 μg/ml) (Sigma-Aldrich) in the presence or absence of quercetin (25, 50 μM) (Solarbio), celecoxib (PTGS2-selective inhibitor) (10 μM) (MCE, HY-14398), and rebamipide (PTGS2 agonists) (100 nM) (MCE, HY-B0360) for 24 h.

### Reverse Transcriptase Polymerase Chain Reaction Analysis

Total RNA of HUVECs was isolated using E.Z.N.A.® HP Total RNA Kit (Omega, USA) according to the manufacturer's instructions. Nanodrop 2000 (Thermo Scientific, USA) was used to detect the concentration and purity of RNA, and complementary DNA (cDNA) was synthesized according to the manufacturer's instructions of PrimeScriptTM RT reagent Kit (Toyobo, Japan). The messenger RNA (mRNA) expression levels of vascular cell adhesion molecule 1 (VCAM-1) and intracellular adhesion molecule 1 (ICAM-1) were detected using quantitative real-time polymerase chain reaction (RT-qPCR) on the BioRad CFX96 (Bio-Rad, USA) with the Fast SYBR® Green PCR Master Mix-PE (Applied Biosystem, USA). Relative expressions were calculated as 2^−Δ*Ct*^ using glyceraldehyde 3-phosphate dehydrogenase (GAPDH) as a reference gene. Primers are listed in Supplementary Table 4.

### Adhesion Assay

About 2.5 × 10^5^/well HUVECs were seeded in 12-well plates overnight and treated with LPS (10 μg/ml) in the presence or absence of quercetin, celecoxib, and rebamipide for 24 h. THP-1 cells (5 × 10^5^ cells/mL) were stained with 5 μM BCECF-AM (Beyotime) in the dark for 30 min and cocultured with HUVECs at 37°C for 60 min. Non-adherent cells were removed and washed with phosphate-buffered saline (PBS) for twice. The attached cells were observed with an inverted fluorescence microscope, and the pictures were captured in three random fields per well at 400 × magnification. The number of adherent cells was quantified by ImageJ software.

### Statistical Analysis

All statistical analyses were performed on SPSS version 23.0 (SPSS, USA) and GraphPad Prism 8.0 (GraphPad Software, USA). Statistical significance of the differences was analyzed with in independent *t*-test or Mann–Whitney U-test. *P* < 0.05 (two-tailed) was considered statistically significant.

## Results

### Effective Components and Targets of XFZYD

A total of 129 compounds and 256 target proteins in 11 kinds of herbs were identified in XFZYD through the TCMSP database with the criteria of OB ≥30%, HL ≥4, and DL ≥0.18 (Supplementary Table 1).

### ASCVD-Related Targets

**Figure 1:**
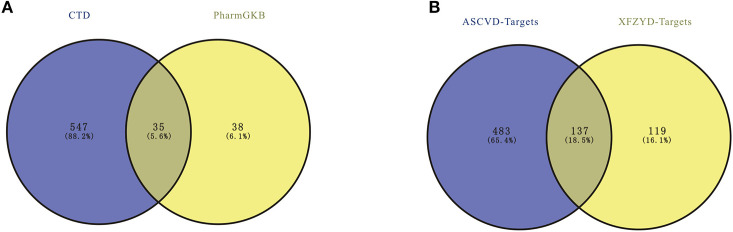
**(A)** The atherosclerosis cardiovascular disease (ASCVD)-related targets. **(B)** The overlapped targets of ASCVD and Xuefu Zhuyu decoration (XFZYD).

ASCVD-related therapeutic targets were retrieved in two databases, including 73 in PharmGKB and 582 in CTD. After removing the repeated targets, a total of 620 ASCVD-related therapeutic targets were identified (Supplementary Table 2), 137 of which were overlapped with targets of XFZYD (Figure 1).

### Compound–Target Network of XFZYD for the Treatment of ASCVD

**Figure 2:**
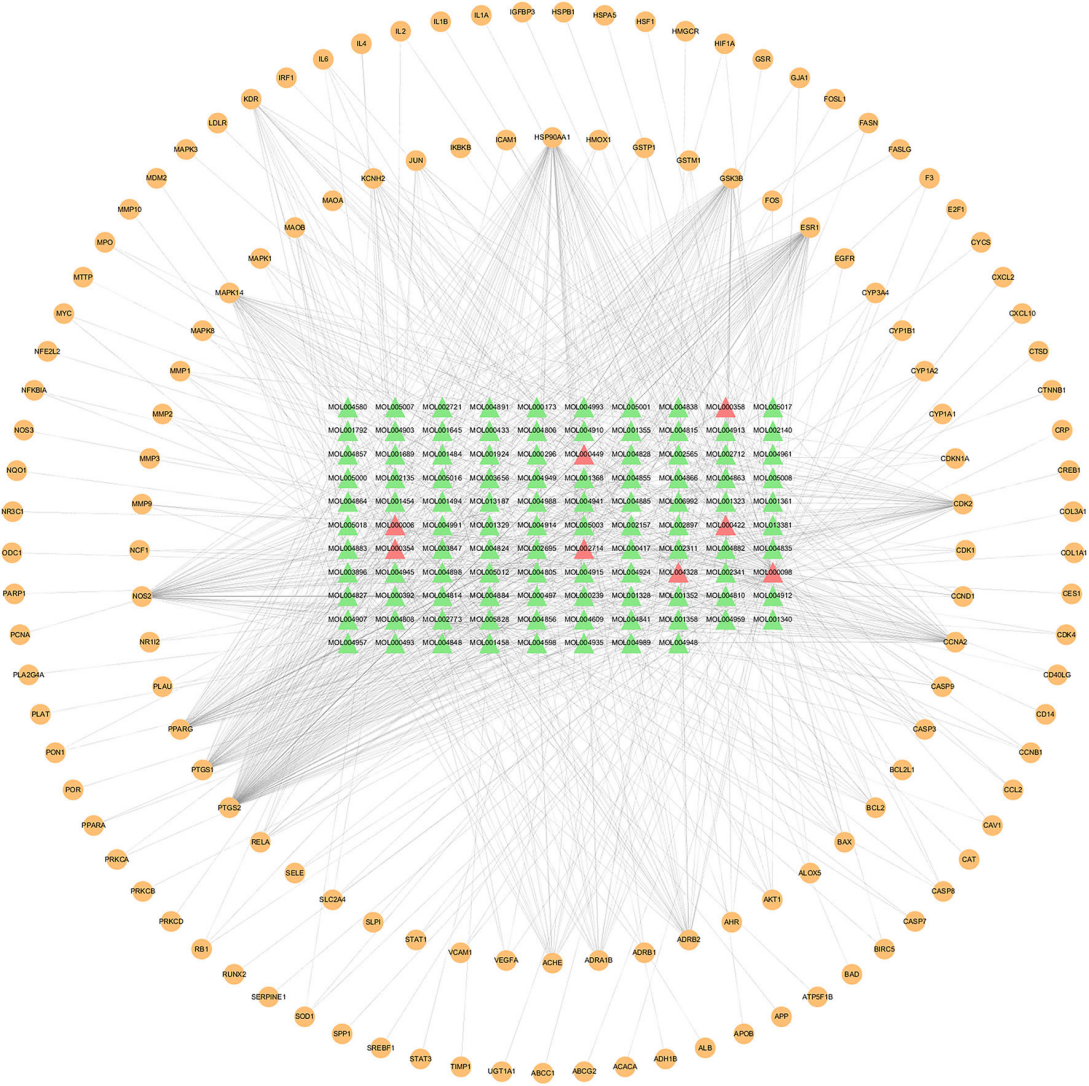
Compounds–targets network of Xuefu Zhuyu decoration (XFZYD) in treating ASCVD. Orange ellipse nodes represent potential therapeutic targets, green triangle nodes represent effective compounds, and red triangle nodes represent the effective compound originated from multiple herbs.

**Figure 3:**
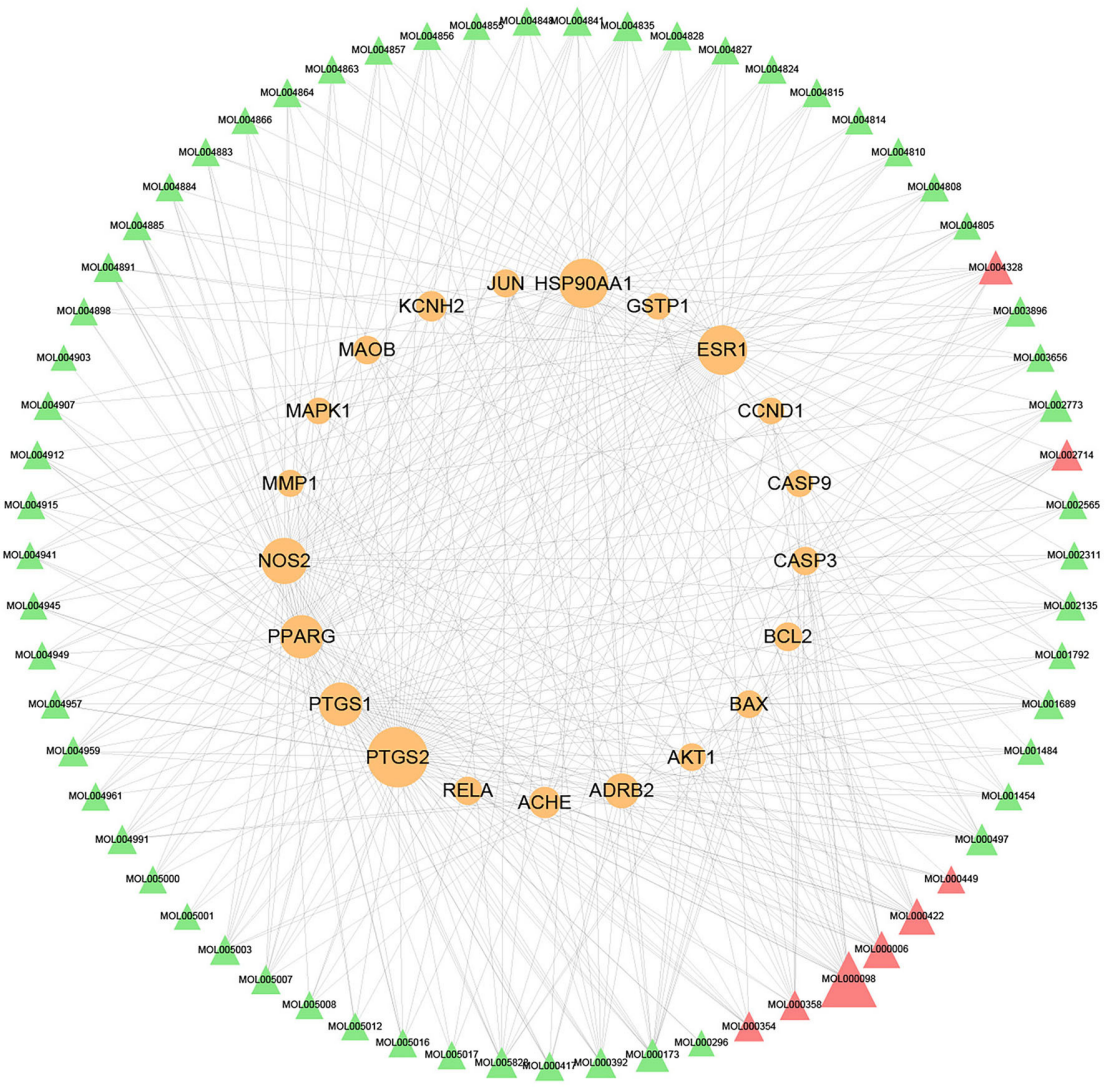
Topological compounds–targets network of Xuefu Zhuyu decoration (XFZYD). Orange ellipse nodes represent potential therapeutic targets, and green and red triangle nodes represent effective compounds. The size of node is proportional to the value of the degree centrality by topology analysis.

The compound–target network was constructed to elaborate the multiplex interplay between compounds and their related targets of XFZYD at a systematic perspective. The compound–target network consists of 245 nodes (108 active components and 137 candidate therapeutic targets) and 945 edges (Figure 2). The compound quercetin has 90 targets, suggesting that it may be critical in the treatment of atherosclerosis (Table 1). Topological analysis was adopted to determine the core targets and compounds of XFZYD in the treatment of ASCVD with the screening criteria “DC ≥ 3, BC ≥ 0.000348, and CC ≥ 0.3754.” The topological network consists of 86 nodes (65 active components and 21 component targets) and 441 edges (Figure 3). From this result, it can be inferred that these high-degree compounds are likely to be the core pharmacodynamic substances in the XFZYD. Besides, the compound–target network illustrated the multicomponent, multitarget characteristics of XFZYD.

**Table 1:** The characteristics of effective compounds in Xuefu Zhuyu decoration (XFZYD).

**Mol ID**	**Compounds**	**OB (%)**	**DL**	**HL**	**Herb**	**Target number**
MOL000006	Luteolin	36.16	0.25	15.94	*Flos Carthami* *Radix Platycodonis*	35
MOL000098	Quercetin	46.43	0.28	14.4	*Radix Bupleuri* *Flos Carthami* *Radix Achyranthis* *Bidentatae* *Radix Glycyrrhizae*	90
MOL000173	Wogonin	30.68	0.23	17.75	*Radix Achyranthis* *Bidentatae*	24
MOL000239	Jaranol	50.83	0.29	15.5	*Radix Glycyrrhizae*	5
MOL000296	Hederagenin	36.91	0.75	5.35	*Semen Persicae*	4
MOL000354	Isorhamnetin	49.6	0.31	14.34	*Radix Bupleuri* *Radix Glycyrrhizae*	14
MOL000358	Beta-sitosterol	36.91	0.75	5.36	*Radix Paeoniae Rubra* *Radix Angelicae Sinensis* *Flos Carthami* *Radix Achyranthis* *Bidentatae* *Semen Persicae* *Fructus Aurantii*	14
MOL000392	Formononetin	69.67	0.21	17.04	*Radix Glycyrrhizae*	16
MOL000417	Calycosin	47.75	0.24	17.1	*Radix Glycyrrhizae*	11
MOL000422	Kaempferol	41.88	0.24	14.74	*Radix Bupleuri* *Radix Glycyrrhizae* *Flos Carthami* *Radix Achyranthis* *Bidentatae*	33
MOL000433	FA	68.96	0.71	24.81	*Rhizoma Chuanxiong*	2
MOL000449	Stigmasterol	43.83	0.76	5.57	*Radix Bupleuri* *Radix Paeoniae Rubra* *Radix Angelicae* *Sinensis* *Radix Rehmanniae* *Flos Carthami* *Radix Achyranthis* *Bidentatae*	8
MOL000493	Campesterol	37.58	0.71	4.71	*Semen Persicae*	3
MOL000497	Licochalcone a	40.79	0.29	16.2	*Radix Glycyrrhizae*	19
MOL001323	Sitosterol alpha1	43.28	0.78	5.64	*Semen Persicae*	1
MOL001328	2,3-Didehydro GA70	63.29	0.5	7.62	*Semen Persicae*	2
MOL001329	2,3-Didehydro GA77	88.08	0.53	7.6	*Semen Persicae*	1
MOL001340	GA120	84.85	0.45	8.4	*Semen Persicae*	1
MOL001352	GA54	64.21	0.53	10.19	*Semen Persicae*	2
MOL001355	GA63	65.54	0.54	9.85	*Semen Persicae*	1
MOL001358	Gibberellin 7	73.8	0.5	9.79	*Semen Persicae*	2
MOL001361	GA87	68.85	0.57	8.76	*Semen Persicae*	1
MOL001368	3-O-p-Coumaroylquinic acid	37.63	0.29	5.15	*Semen Persicae*	3
MOL001454	Berberine	36.86	0.78	6.57	*Radix Achyranthis* *Bidentatae*	7
MOL001458	Coptisine	30.67	0.86	9.33	*Radix Achyranthis* *Bidentatae*	5
MOL001484	Inermine	75.18	0.54	11.72	*Radix Glycyrrhizae*	5
MOL001494	Mandenol	42	0.19	5.39	*Rhizoma Chuanxiong*	2
MOL001645	Linoleyl acetate	42.1	0.2	7.48	*Radix Bupleuri*	2
MOL001689	Acacetin	34.97	0.24	17.25	*Radix Platycodonis*	14
MOL001792	DFV	32.76	0.18	17.89	*Radix Glycyrrhizae*	6
MOL001924	Paeoniflorin	53.87	0.79	13.88	*Radix Paeoniae Rubra*	2
MOL002135	3,5,6,7-Tetramethoxy−2-(3,4,5-Trimethoxyphenyl) chromone	40.6	0.51	4.39	*Rhizoma Chuanxiong*	13
MOL002140	Perlolyrine	65.95	0.27	12.62	*Rhizoma Chuanxiong*	1
MOL002157	Wallichilide	42.31	0.71	6.85	*Rhizoma Chuanxiong*	2
MOL002311	Glycyrol	90.78	0.67	9.85	*Radix Glycyrrhizae*	8
MOL002341	Hesperetin	70.31	0.27	15.78	*Fructus Aurantii*	3
MOL002565	Medicarpin	49.22	0.34	8.46	*Radix Glycyrrhizae*	9
MOL002695	Lignan	43.32	0.65	14.88	*Flos Carthami*	2
MOL002712	6-Hydroxykaempferol	62.13	0.27	14.29	*Flos Carthami*	5
MOL002714	Baicalein	33.52	0.21	16.25	*Radix Achyranthis* *Bidentatae* *Flos Carthami* *Radix Paeoniae Rubra*	18
MOL002721	Quercetagetin	45.01	0.31	13.82	*Flos Carthami*	3
MOL002773	Beta-carotene	37.18	0.58	4.36	*Flos Carthami*	21
MOL002897	Epiberberine	43.09	0.78	6.1	*Radix Achyranthis* *Bidentatae*	4
MOL003656	Lupiwighteone	51.64	0.37	15.63	*Radix Glycyrrhizae*	9
MOL003847	Inophyllum E	38.81	0.85	15.51	*Radix Achyranthis* *Bidentatae*	4
MOL003896	7-Methoxy-2- methyl isoflavone	42.56	0.2	16.89	*Radix Glycyrrhizae*	15
MOL004328	Naringenin	59.29	0.21	16.98	*Radix Glycyrrhizae* *Fructus Aurantii*	26
MOL004580	cis-Dihydroquercetin	66.44	0.27	14.51	*Radix Platycodonis*	3
MOL004598	3,5,6,7-Tetramethoxy-2- (3,4,5-Trimethoxyphenyl) Chromone	31.97	0.59	15.54	*Radix Bupleuri*	3
MOL004609	Areapillin	48.96	0.41	16.52	*Radix Bupleuri*	3
MOL004805	(2S)-2-[4-Hydroxy-3- (3-methylbut-2-enyl)phenyl]−8,8-Dimethyl-2,3- dihydropyrano[2,3-f]chromen−4-one	31.79	0.72	14.82	*Radix Glycyrrhizae*	7
MOL004806	Euchrenone	30.29	0.57	15.89	*Radix Glycyrrhizae*	4
MOL004808	Glyasperin B	65.22	0.44	16.1	*Radix Glycyrrhizae*	10
MOL004810	Glyasperin F	75.84	0.54	15.64	*Radix Glycyrrhizae*	10
MOL004814	Isotrifoliol	31.94	0.42	7.91	*Radix Glycyrrhizae*	8
MOL004815	(E)-1-(2,4-dihydroxyphenyl)−3-(2,2-Dimethylchromen-6-yl) prop-2-en-1-one	39.62	0.35	16.16	*Radix Glycyrrhizae*	10
MOL004824	(2S)-6-(2,4-dihydroxyphenyl)−2-(2-hydroxypropan-2-yl)-4 -methoxy-2,3-dihydrofuro [3,2-g]chromen-7-one	60.25	0.63	4.31	*Radix Glycyrrhizae*	10
MOL004827	Semilicoisoflavone B	48.78	0.55	17.02	*Radix Glycyrrhizae*	8
MOL004828	Glepidotin A	44.72	0.35	16.09	*Radix Glycyrrhizae*	11
MOL004835	Glypallichalcone	61.6	0.19	17.01	*Radix Glycyrrhizae*	13
MOL004838	8-(6-Hydroxy-2-benzofuranyl)−2,2-dimethyl-5-chromenol	58.44	0.38	8.71	*Radix Glycyrrhizae*	4
MOL004841	Licochalcone B	76.76	0.19	17.02	*Radix Glycyrrhizae*	11
MOL004848	Licochalcone G	49.25	0.32	15.75	*Radix Glycyrrhizae*	10
MOL004855	Licoricone	63.58	0.47	16.37	*Radix Glycyrrhizae*	6
MOL004856	Gancaonin A	51.08	0.4	16.82	*Radix Glycyrrhizae*	8
MOL004857	Gancaonin B	48.79	0.45	16.49	*Radix Glycyrrhizae*	10
MOL004863	3-(3,4-Dihydroxyphenyl)-5,7- dihydroxy-8-(3-methylbut-2-enyl) chromone	66.37	0.41	15.81	*Radix Glycyrrhizae*	9
MOL004864	5,7-Dihydroxy-3-(4-methoxyphenyl)−8-(3-methylbut-2-enyl)chromone	30.49	0.41	14.99	*Radix Glycyrrhizae*	10
MOL004866	2-(3,4-Dihydroxyphenyl)-5,7- dihydroxy-6-(3-methylbut-2-enyl) chromone	44.15	0.41	16.77	*Radix Glycyrrhizae*	6
MOL004882	Licocoumarone	33.21	0.36	9.66	*Radix Glycyrrhizae*	5
MOL004883	Licoisoflavone	41.61	0.42	16.09	*Radix Glycyrrhizae*	9
MOL004884	Licoisoflavone B	38.93	0.55	15.73	*Radix Glycyrrhizae*	8
MOL004885	Licoisoflavanone	52.47	0.54	15.67	*Radix Glycyrrhizae*	10
MOL004891	Shinpterocarpin	80.3	0.73	6.5	*Radix Glycyrrhizae*	12
MOL004898	(E)-3-[3,4-Dihydroxy-5- (3-methylbut-2-enyl)phenyl]−1-(2,4-dihydroxyphenyl)prop−2-en-1-one	46.27	0.31	15.24	*Radix Glycyrrhizae*	8
MOL004903	Liquiritin	65.69	0.74	17.96	*Radix Glycyrrhizae*	3
MOL004907	Glyzaglabrin	61.07	0.35	21.2	*Radix Glycyrrhizae*	10
MOL004910	Glabranin	52.9	0.31	16.24	*Radix Glycyrrhizae*	5
MOL004912	Glabrone	52.51	0.5	16.09	*Radix Glycyrrhizae*	10
MOL004913	1,3-Dihydroxy-9-methoxy-6- benzofurano[3,2-c]chromenone	48.14	0.43	8.87	*Radix Glycyrrhizae*	7
MOL004914	1,3-Dihydroxy-8,9-dimethoxy-6-benzofurano[3,2-c]chromenone	62.9	0.53	9.32	*Radix Glycyrrhizae*	6
MOL004915	Eurycarpin A	43.28	0.37	17.1	*Radix Glycyrrhizae*	9
MOL004924	(–)-Medicocarpin	40.99	0.95	13.2	*Radix Glycyrrhizae*	2
MOL004935	Sigmoidin-B	34.88	0.41	14.49	*Radix Glycyrrhizae*	4
MOL004941	(2R)-7-hydroxy-2-(4-hydroxyphenyl) chroman-4-one	71.12	0.18	18.09	*Radix Glycyrrhizae*	6
MOL004945	(2S)-7-Hydroxy-2-(4-hydroxyphenyl)−8-(3-methylbut-2-enyl)chroman-4-one	36.57	0.32	17.95	*Radix Glycyrrhizae*	7
MOL004948	Isoglycyrol	44.7	0.84	6.69	*Radix Glycyrrhizae*	4
MOL004949	Isolicoflavonol	45.17	0.42	15.55	*Radix Glycyrrhizae*	8
MOL004957	HMO	38.37	0.21	16.56	*Radix Glycyrrhizae*	11
MOL004959	1-Methoxyphaseollidin	69.98	0.64	9.53	*Radix Glycyrrhizae*	14
MOL004961	Quercetin der.	46.45	0.33	16.61	*Radix Glycyrrhizae*	9
MOL004988	Kanzonol F	32.47	0.89	9.98	*Radix Glycyrrhizae*	2
MOL004989	6-Prenylated eriodictyol	39.22	0.41	16.52	*Radix Glycyrrhizae*	4
MOL004991	7-Acetoxy-2-methylisoflavone	38.92	0.26	17.49	*Radix Glycyrrhizae*	12
MOL004993	8-Prenylated eriodictyol	53.79	0.4	15.7	*Radix Glycyrrhizae*	3
MOL005000	Gancaonin G	60.44	0.39	16.13	*Radix Glycyrrhizae*	8
MOL005001	Gancaonin H	50.1	0.78	16.64	*Radix Glycyrrhizae*	5
MOL005003	Licoagrocarpin	58.81	0.58	9.45	*Radix Glycyrrhizae*	14
MOL005007	Glyasperins M	72.67	0.59	15.57	*Radix Glycyrrhizae*	12
MOL005008	Glycyrrhiza flavonol A	41.28	0.6	13.71	*Radix Glycyrrhizae*	8
MOL005012	Licoagroisoflavone	57.28	0.49	19.64	*Radix Glycyrrhizae*	8
MOL005016	Odoratin	49.95	0.3	16.35	*Radix Glycyrrhizae*	10
MOL005017	Phaseol	78.77	0.58	9.64	*Radix Glycyrrhizae*	9
MOL005018	Xambioona	54.85	0.87	14.5	*Radix Glycyrrhizae*	3
MOL005828	Nobiletin	61.67	0.52	16.2	*Fructus Aurantii*	17
MOL006992	(2R,3R)-4-Methoxyl-distylin	59.98	0.3	15.08	*Radix Paeoniae Rubra*	5
MOL013187	Cubebin	57.13	0.64	12.4	*Radix Bupleuri*	4
MOL013381	Marmin	38.23	0.31	4.68	*Fructus Aurantii*	2

### KEGG Enrichment Analysis

**Figure 4:**
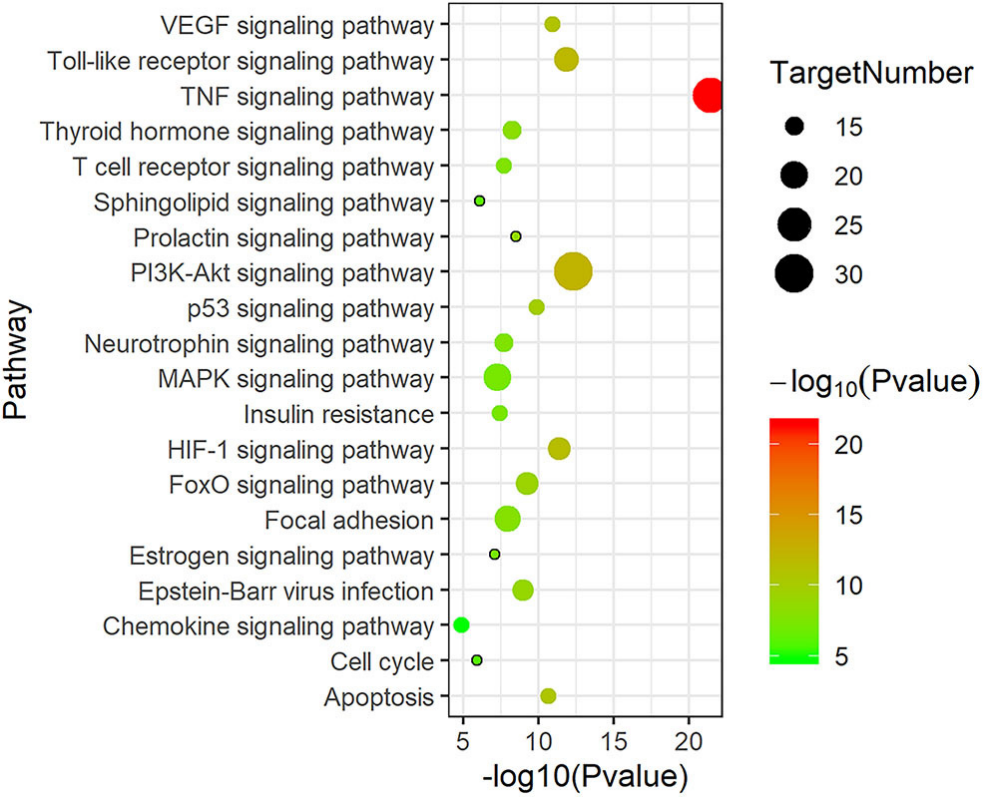
The top 20 Kyoto Encyclopedia of Genes and Genomes (KEGG) pathway enrichment analyses of 137 target proteins (*P* < 0.05).

KEGG pathway enrichment analysis was performed to elucidate related pathways of the 137 candidate therapeutic targets. The representative top 20 pathways based on the number of enriched genes as well as *P*-value are shown in Figure 4. These key targets were closely related to the tumor necrosis factor (TNF) signaling pathway, PI3K-Akt signaling pathway, vascular endothelial growth factor (VEGF) signaling pathway, Toll-like receptor signaling pathway, etc., participating in the process of atherosclerotic plaque formation, such as oxidative stress, inflammatory response, angiogenesis, etc.

### Targets in the Intersection With GEO Database

With the analysis of GSE71226 microarray data of atherosclerosis, 673 differentially expressed genes were identified, among which 133 were upregulated and 540 were downregulated in the atherosclerosis group (Supplementary Table 3). There were six targets in the intersection with 137 candidate therapeutic targets of XFZYD, which included upregulated PTGS2, MMP9, and BCL2L1 and downregulated JUN, VEGFA, and CXCL2 in the atherosclerosis group (Table 2).

**Table 2:** Differentially expressed genes (DEGs) that overlapped with potential therapeutic targets of Xuefu Zhuyu decoration (XFZYD).

**Group**	**Gene symbol**	**Gene title**	***P*-value**	**logFC**
Upregulated genes	BCL2L1	BCL2-like 1	0.0163396	2.38
	MMP9	Matrix metallopeptidase 9	0.0187166	2.27
	PTGS2	Prostaglandin-endoperoxide synthase 2	0.0374968	1.75
Downregulated genes	CXCL2	C-X-C motif chemokine ligand 2	0.00806	−1.59
	JUN	Jun proto-oncogene, AP-1 transcription factor subunit	0.0008752	−1.99
	VEGFA	Vascular endothelial growth factor A	0.0011307	−1.69

### Molecular Docking

To confirm the binding capacity between active compounds and key therapeutic targets, molecular docking using Schrodinger was performed. The target proteins' degrees ranked top 5 in compound–target network topology analysis, and six above DEGs overlapped with candidate therapeutic targets were selected as docking objects. The docking results are listed in Supplementary Table 5. There were eight targets binding to the quercetin in these 10 docking targets, indicating that quercetin might be the main component of XFZYD to exert antiatherosclerosis efficiency.

### Quercetin Suppressed LPS-Induced Attachment of HUVECs

**Figure 5:**
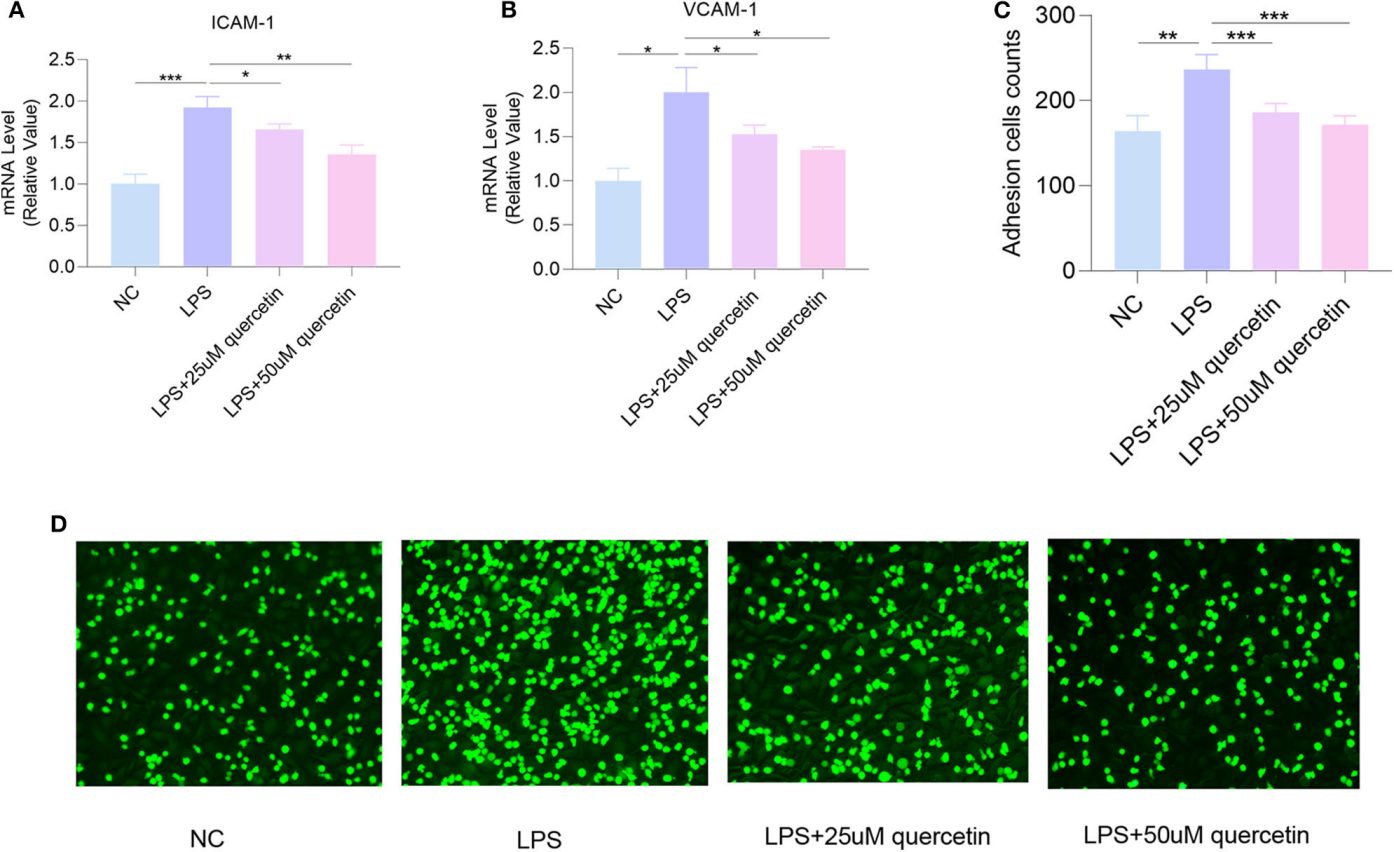
Quercetin attenuates LPS-induced attachment of human umbilical vein endothelial cells (HUVECs). The messenger RNA (mRNA) levels of **(A)** intracellular adhesion molecule 1 (ICAM-1) and **(B)** vascular cell adhesion molecule 1 (VCAM-1). **(C,D)** The number of THP-1 cells attached to HUVECs. Data are listed as mean ± SD of at least three independent experiments. NC, negative control; LPS, lipopolysaccharide. *P < 0.05, **P < 0.01, ***P < 0.001.

The mRNA levels of VCAM-1 and ICAM-1 in HUVECs were significantly increased by exposure to 10 μg/ml LPS (both *P* < 0.0001), which were decreased by quercetin (25 and 50 μM) (Figures 5A,B). Moreover, quercetin could significantly inhibit LPS-induced attachment of monocytes to HUVECs (Figures 5C,D), suggesting that quercetin may alleviate endothelial dysfunction by reducing adhesion of endothelial cells.

### Quercetin Inhibited LPS-Induced Elevation of Pro-inflammatory Cytokines

**Figure 6:**
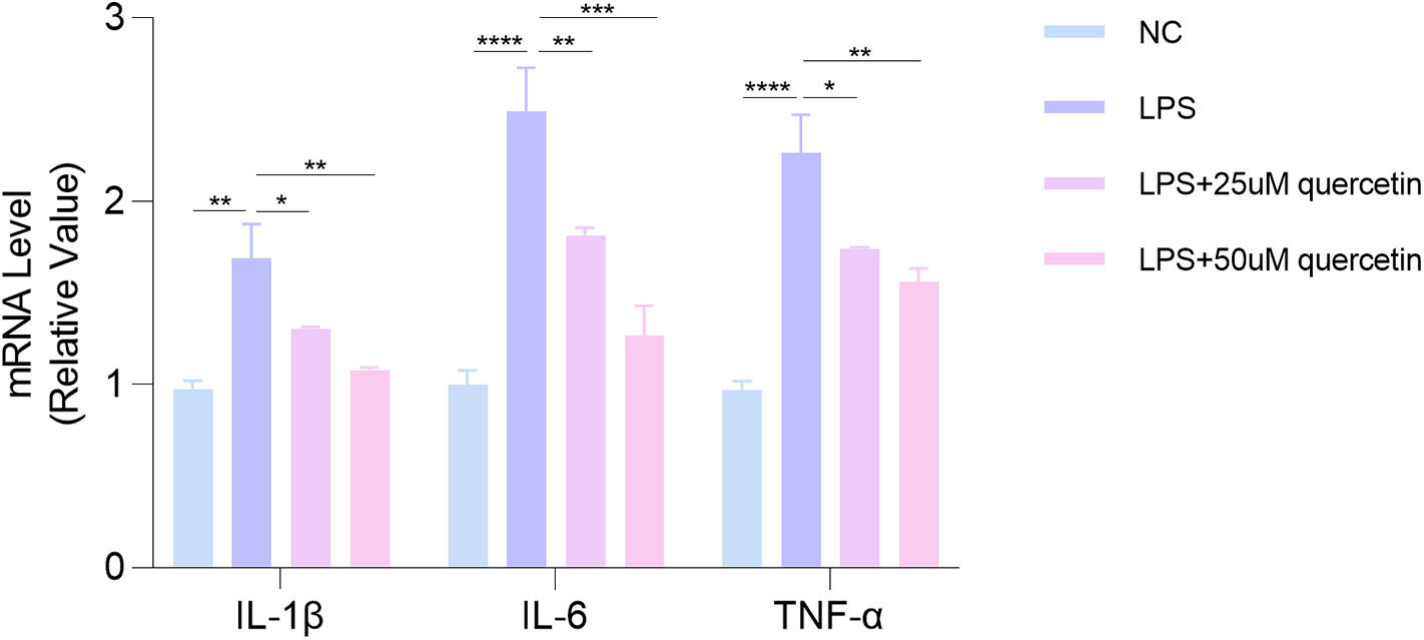
Quercetin attenuates LPS-induced the elevation of pro-inflammatory cytokines of human umbilical vein endothelial cells (HUVECs). Data are listed as mean ± SD of at least three independent experiments. NC, negative control; LPS, lipopolysaccharide. *P < 0.05, **P < 0.01, ***P < 0.001, ****P < 0.0001.

In order to study the effect of quercetin on pro-inflammatory cytokines expression, HUVECs were stimulated with LPS in the presence or absence of quercetin (25, 50 μM) for 24 h. As shown in Figure 6, LPS treatment significantly increased the mRNA levels of IL-1β (*P* < 0.001), IL-6 (*P* < 0.0001), and TNF-α (*P* < 0.0001) in HUVECs, which were reversed by quercetin (25, 50 μM). These results suggested a strong inhibitory effect of quercetin on pro-inflammatory cytokines.

### PTGS2 Might Be a Therapeutic Target of XFZYD

**Figure 7:**
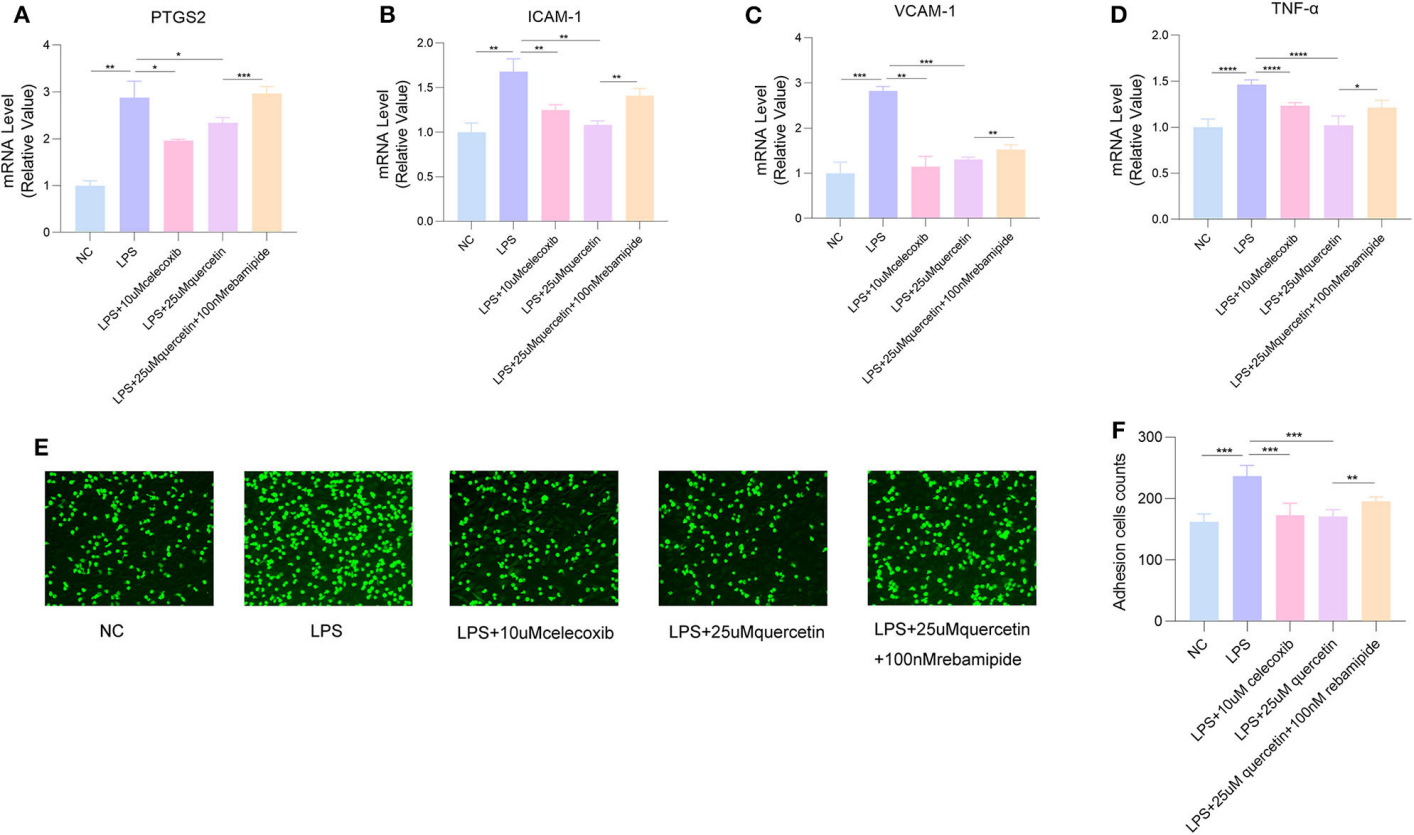
Celecoxib inhibited LPS-induced attachment of human umbilical vein endothelial cells (HUVECs), while rebamipide attenuated the inhibition effect of quercetin. The messenger RNA (mRNA) levels of **(A)** PTGS2, **(B)** intracellular adhesion molecule 1 (ICAM-1), **(C)** vascular cell adhesion molecule 1 (VCAM-1), and **(D)** tumor necrosis factor alpha (TNF-α). **(E,F)** The number of THP-1 cells attached to HUVECs. Data are listed as mean ± SD of at least three independent experiments. NC, negative control; LPS, lipopolysaccharide. *P < 0.05, **P < 0.01, ***P < 0.001, ****P < 0.0001.

The inhibitor of PTGS2 celecoxib could decrease the expressions of VCAM-1, ICAM-1, and TNF-α in HUVECs induced by LPS, and significantly inhibit LPS-induced attachment of monocytes to HUVECs, while the agonists rebamipide attenuated the inhibition effect of quercetin on adhesion molecules VCAM-1, ICAM-1, and pro-inflammatory cytokine TNF-α as well as the number of monocytes attached to endothelial cells (Figure 7), suggesting that high-expression PTGS2 might be the target of quercetin and inhibition of PTGS2 may improve endothelial dysfunction by reducing adhesion of endothelial cells.

## Discussion

ASCVD is a kind of complex and multifactorial disease and remains the leading cause of death worldwide (20). TCM has characteristics of multicomponent and multitarget, which can affect different biological processes to control symptoms and solve the fundamental problems.

In the present study, the effective compounds and candidate therapeutic targets in XFZYD for the treatment of ASCVD were 108 and 137, respectively. Moreover, 56.2% of 137 candidate therapeutic targets of XFZYD could be overlapped by at least two effective compounds, which demonstrated the effective compounds in XFZYD worked against ASCVD through a multitarget synergistic way. In addition, 94.44% of 108 effective compounds acted on at least two candidate therapeutic targets. Besides, quercetin was contained in four herbs (*Radix Bupleuri, Flos Carthami, Radix Achyranthis Bidentatae*, and *Radix Glycyrrhizae*), luteolin was contained in two herbs (*Flos Carthami* and *Radix Platycodonis*), and kaempferol was contained in four herbs (*Radix Bupleuri, Radix Glycyrrhizae, Flos Carthami*, and *Radix Achyranthis Bidentatae*). They acted on 90, 35, and 33 candidate therapeutic targets against ASCVD, respectively, suggesting that XFZYD is a combination of multiple herbs, multiple compounds, and multiple targets in the treatment of ASCVD. Since quercetin has the most predicted targets and was identified as the main component of XFZYD by UHPLC-ESI-IT-TOF-MS (9), it was selected as the tested effective compound.

LPS was considered as an important risk factor contributing to endothelial dysfunction, which was usually known as one of the early hallmarks of atherosclerosis (21, 22). Sequentially, monocyte adhesion to endothelial cells was a crucial step in the early stages of atherosclerosis development, in which ICAM-1 and VCAM-1 were crucially involved. Therefore, preventing endothelial injury has aroused considerable attention as a potential therapeutic target for cardiovascular disease treatment. In this study, quercetin could inhibit LPS-induced attachment of monocytes to HUVECs by downregulating expressions of VCAM-1 and ICAM-1 *in vitro* as well as suppress the expression of pro-inflammatory cytokines, which demonstrated the ability of quercetin to ameliorate endothelial dysfunction. Coincident with our results, quercetin was reported to remarkably suppress endothelial dysfunction and significantly attenuate atherosclerotic lesion progression in high-fat diet (HFD)-fed ApoE^−/−^ mice (23). Likewise, it was demonstrated that quercetin had an inhibitory action on the expression of pro-inflammatory cytokines in hypercholesterolemic diet (HCD)-fed rats (24). Quercetin also exhibited the therapeutic ability through restraining endothelial dysfunction and vascular inflammation produced by LPS *in vivo* (25). Furthermore, previous studies have shown the therapeutic application of quercetin in the treatment of other diseases. Nanocapsulated quercetin significantly reduced the incidence of hepatocellular carcinoma in rats and decreased the production of TNF-α and IL-6 in liver induced by diethyl nitrosamine (26). Supplementation with quercetin were proved to improve hyperglycemia, dyslipidemia, and antioxidant status in type 2 diabetes (27). Quercetin also has protective effects on neuronal cell death, Aβ-induced oxidative stress, and memory degradation (28). Taken together, our prediction results were in line with previous reports and confirmed the reliability of the prediction on effective compounds of XFZYD against atherosclerosis.

Besides, KEGG results also suggested that XFZYD might reduce the inflammatory response in atherosclerosis principally by affecting TNF signaling pathway and Toll-like receptor (TLR) signaling pathway. As for TNF signaling pathway, its downstream gene IL-1β activates the nuclear factor-κB (NF-κB) signaling pathway (29), induces the production of various pro-inflammatory cytokines such as TNF-α and IL-6, and positively regulates the further activation of NF-κB, resulting in an inflammatory cascade amplification effect (30). TLRs were the well-defined pattern recognition receptors of immune system, participating in the chronic inflammation and immune response in atherosclerosis (31). TLRs engagement with their ligands stimulated pro-inflammatory cytokine production and foam cell generation, mediating the occurrence and development of coronary atherosclerotic plaque by regulating inflammation and immune response (32). Activation of the TLR signaling pathway lead to the production of multiple pro-inflammatory cytokines (IL-6, TNF-α), accelerating the pathological process of atherosclerosis (33, 34). In this study, the effective compound quercetin possessed a restrain function on pro-inflammatory cytokines produced by LPS-stimulated endothelial cells, which partly explained the therapeutic effect of XFZYD on atherosclerosis by inhibiting inflammatory pathways.

As for the predicted therapeutic targets, PTGS2 was selected for the verification, which was reported to be involved in the early atherosclerotic process, and PTGS2 was highly expressed in atherosclerotic lesions from both human (35, 36) and animals (37, 38). PTGS2 promoted early atherosclerotic lesion formation in low-density lipoprotein receptor deficient (LDLR–/–) mice *in vivo* (37). Selective inhibitor of PTGS2 celecoxib prevented the development of atherosclerotic lesions in the proximal aortas from ApoE–/– mice by reducing the expressions of ICAM-1 and VCAM-1 (39). Interestingly, Metzner et al. believed that PTGS2 inhibitor had a dual effect on atherosclerosis: promoting the occurrence and development of atherosclerosis in the early stage of atherosclerosis, but playing a protective role against atherosclerosis in the late stage of atherosclerosis (40). In present study, we found that the selective PTGS2 inhibitor celecoxib could reduce the expression of adhesion molecules VCAM-1 and ICAM-1 as well as pro-inflammatory cytokine TNF-α on HUVECs induced by LPS and decrease the amount of monocytes attached to HUVECs, while rebamipide (PTGS2 agonists) weakened the inhibitory effect of quercetin on cell adhesion of HUVECs and pro-inflammatory cytokine, implying that quercetin might play an antiatherosclerosis role by restraining PTGS2 to improve the dysfunction of vascular endothelial cells.

However, there were still some shortcomings in this research. Since the pathological development of atherosclerosis involves complex pathological processes, the mechanism predicted above of XFZYD in treating atherosclerosis still needs to be supplemented by *in vivo* and *in vitro* experiments. Here, we have verified only one effective component *in vitro*, and the other effective components still needed to be confirmed experimentally. In the future, we will continue to explore the mechanisms of XFZYD's other effective components in the treatment of atherosclerotic diseases, in the hope of providing new insights for clinical drug development.

## Conclusions

In this study, the effective components and potential therapeutic targets of XFZYD for ASCVD treatment were explored from the perspective of systemic pharmacology, and the effective component quercetin was proved to protect injured endothelial cells and reduce endothelial inflammatory response *in vitro*. In addition, PTGS2 might be a therapeutic target of quercetin in XFZYD.

## Data Availability Statement

The datasets presented in this study can be found in online repositories. The names of the repository/repositories and accession number(s) can be found in the article/Supplementary Material.

## Author Contributions

FZ designed the experiments. FZ, BL, YX, and XZ wrote the manuscript and conducted the pharmacology information analyses. BL, BJ, and YZ visualized the data. BL and CW completed the *in vitro* experiments. All authors read and approved the final manuscript.

## Conflict of Interest

The authors declare that the research was conducted in the absence of any commercial or financial relationships that could be construed as a potential conflict of interest.
